# Public health round-up

**DOI:** 10.2471/BLT.17.011217

**Published:** 2017-12-01

**Medical evacuation plan for besieged Syrians**

The World Health Organization (WHO) and its partners prepared a plan last month to evacuate hundreds of patients from the eastern Ghouta region of the Syrian Arab Republic to medical facilities in other parts of the country. This child is being measured by a health professional to determine whether she has severe acute malnutrition during a United Nations humanitarian mission to the region, where up to 400 000 people face deteriorating conditions.

http://www.emro.who.int/syr/syria-news/who-calls-for-immediate-and-unimpeded-access-to-save-lives-in-eastern-ghouta-syrian-arab-republic.html

**Figure:**
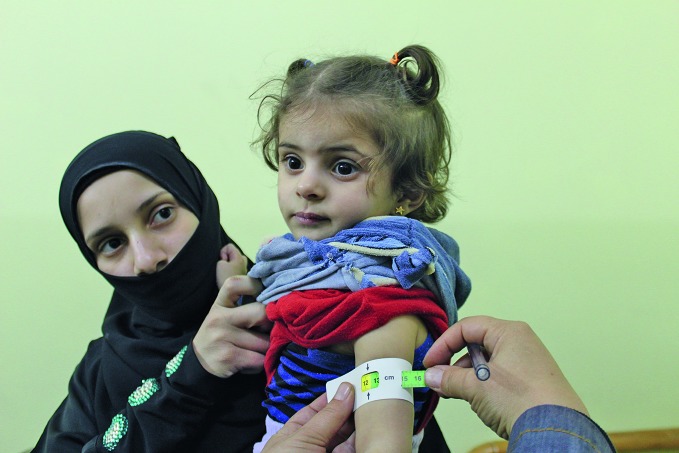
OCHA/Linda Tom

## New vision for WHO

The Word Health Organization’s (WHO) new Director-General Dr Tedros Adhanom Ghebreyesus, who took office on 1 July, unveiled his vision for the future course of the organization’s work in countries throughout the world.

“The world expects WHO to do three things: promote health, keep the world safe and serve the vulnerable. That’s our mission,” he told a meeting of staff from headquarters, regional and country offices last month.

The new mission statement is supported by proposed strategic objectives, each with targets outlined in the draft 13th General Programme of Work (GPW) for 2019–2023.

The draft GPW was posted on the internet for public consultation from 1 to 15 November and discussed on 22 and 23 November at a special session of the WHO Executive Board.

The GPW is being prepared over a period of five months, rather than the usual 18 months, and its strategic objectives focus on outcomes and impact rather than output and process. 

The proposed objectives are: universal health coverage with a target to extend health coverage to 1 billion more people; global health security, with a target to make 1 billion people safer, and progress towards the sustainable development goals with a target to improve the lives of 1 billion people.

In addition, the GPW focuses on four areas: noncommunicable diseases including mental health; climate change with an emphasis on small island states; human capital; and antimicrobial resistance.

The Director-General also presented a WHO transformation plan to staff. “This [plan] will be the mechanism for making WHO the organization it needs to be to deliver on our new strategic plan,” he said.

More than 260 leaders from WHO headquarters, regional and country offices gathered in Geneva from 30 October to 1 November to discuss the two drafts. They were joined by the United Nations Development Programme, the International Committee of the Red Cross, GAVI the Vaccine Alliance, the Global Fund to Fight AIDS, Tuberculosis and Malaria and other partners.

Earlier this year, the draft 13th GPW was discussed by officials from WHO Member States at the six regional committee meetings. Next year it will be discussed at the Executive Board meeting on 22–27 January and submitted to the World Health Assembly on 21–26 May for approval.

www.who.int/about/gpw-thirteen-consultation

## Stop using antibiotics in healthy animals

Farmers and the food industry should stop using antibiotics to promote growth and prevent disease in healthy animals, to help prevent the spread of antibiotic resistance in humans, according new WHO recommendations.

The new recommendations and best practice statements, contained in the WHO *Guidelines on use of medically important antimicrobials in food-producing animals* released last month, aim to help preserve the effectiveness of antibiotics that are important for human medicine by reducing their use in livestock.

Over-use and misuse of antibiotics in animals and humans is contributing to the growing threat of antibiotic resistance. Some types of bacteria that cause serious infections in humans have already developed resistance to most or all of the available treatments, and there are few options in the research and development pipeline.

In some countries about 80% of total consumption of antibiotics that are important for human health is in the animal sector, mainly for growth promotion in healthy animals. Antimicrobial-resistant bacteria that emerge in animals can transfer resistance to human pathogens through direct contact between humans and animals, through food and through the environment.

WHO strongly recommends an overall reduction in the use of all classes of medically important antibiotics in food-producing animals, including complete restriction of these antibiotics for growth promotion and disease prevention without diagnosis.

Sick animals should be tested to determine the most effective and prudent antibiotic to treat their specific infection.

Antibiotics used in animals should be selected, starting with those WHO has listed as being “least important” to human health, and not those classified as “highest priority critically important”, which are often the last line, or one of very few treatments available to treat serious bacterial infections in humans.

A systematic review published in *the Lancet planetary health* last month found that interventions that restrict antibiotic use in food-producing animals reduced antibiotic-resistant bacteria in these animals by up to 39%. WHO’s new guidelines are based on these and other research findings.

www.who.int/foodsafety/areas_work/antimicrobial-resistance/cia_guidelines

**Cover photo**

Every year a youth ski camp is organized free of charge by the national ski association Swiss Ski in Lenk, Switzerland. Among the 600 participants who come from across the country are young wheelchair users.

**Figure:**
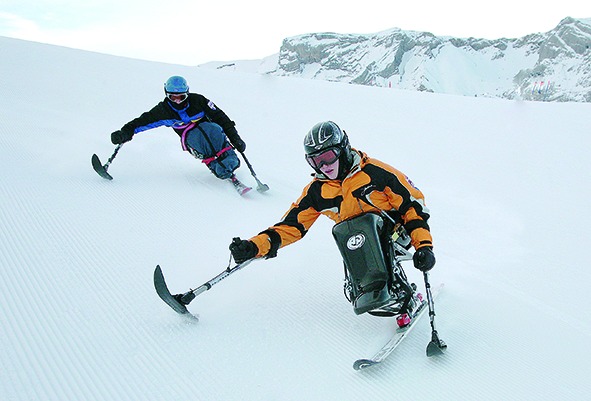
© International Federation for Spina Bifida and Hydrocephalus

## Typhoid vaccine and antimicrobial resistance

The Strategic Advisory Group of Experts (SAGE) on immunization, which advises WHO, recommended a new vaccine, typhoid conjugate vaccine (TCV) for routine use in children over 6 months of age in typhoid endemic countries.

SAGE also called for the introduction of TCV to be prioritized for countries with the highest burden of typhoid disease or of antibiotic resistance to *Salmonella* *Typhi* (*S*.* Typhi*), the bacterium that causes the disease, according to the report of their 17–19 October meeting released this month.

Use of the vaccine should also help to curb the frequent use of antibiotics for treatment of presumed typhoid fever, and thereby help to slow what the experts described as the “alarming increase” in antibiotic resistance in *S*. *Typhi*.

WHO continues to recommend the use of the purified Vi polysaccharide vaccine, known as ViPS, and another vaccine called Ty21a in individuals over 2 years of age or 5 years of age respectively. All three vaccines may be used to control endemic typhoid disease and for outbreak control.

Before and after the introduction of TCV, countries should strengthen the surveillance of typhoid fever and monitor the occurrence of antibiotic resistant strains of *S. Typhi* in endemic and epidemic disease, they said.

New data reveal a heavy burden of typhoid fever in sub-Saharan Africa, in addition to in Asia, and a higher burden of the disease in children under two years of age than previously thought.

Global estimates of the typhoid burden range between 11 and 20 million cases and between about 128 000 and 161 000 typhoid deaths annually.

www.who.int/immunization/policy/sage/en/

## Eliminating lymphatic filariasis

WHO is recommending that countries in which people are affected by lymphatic filariasis, one of the main causes of elephantiasis, switch to a new three-drug treatment regimen in certain settings to boost the global campaign to eliminate the disfiguring disease.

Until now, a two-drug combination of diethylcarbamazine citrate and albendazole has been recommended to treat the parasitic disease.

In countries currently using the combination of diethylcarbamazine and albendazole, WHO is now recommending that a third drug, ivermectin, is added during mass drug administration in settings where the most benefit is expected. Research shows that this addition clears the microfilaria (parasite larvae) more efficiently from the blood than the two-drug regimen and is just as safe.

This is one of several recommendations in WHO guidelines released last month.

The three-drug treatment, known as IDA – named after its three components – is seen as essential for accelerating the global elimination of the disease.

Elimination of lymphatic filariasis is defined as, reducing incidence of infection through mass drug administration to a very low level at which transmission is no longer sustainable.

About 24 countries that currently use the two-drug regimen could benefit from three-drug IDA.

About 856 million people are living in areas where filarial infections are still transmitted.

“Having a more effective combination regimen means we need to overcome poor compliance,” said Dr Jonathan King, scientist in-charge of lymphatic filariasis elimination at WHO’s Department of Control of Neglected Tropical Diseases.

“Now we need wide-ranging communication and delivery approaches to maximize community participation during mass drug administration campaigns. National programmes will have to re-evaluate the performance of existing strategies and engage at-risk communities.”

www.who.int/neglected_diseases/news/WHO_recommends_triple_medicine_therapy_for_LF_elimination

## Violence prevention app

WHO launched a new website application last month where anyone can look up the latest data and information on violence and its prevention in 197 countries and areas of the world.

The application includes more than 3000 studies and provides graphic visualizations of these data covering six areas: homicide, child maltreatment, youth violence, elder abuse, sexual violence and intimate partner violence.

http://apps.who.int/violence-info/

**Looking ahead**

1 December – World AIDS Day

22–27 January 2018 – Executive Board meeting

21–26 May 2018 – World Health Assembly

